# Image quality comparison between a phase-contrast synchrotron radiation breast CT and a clinical breast CT: a phantom based study

**DOI:** 10.1038/s41598-019-54131-z

**Published:** 2019-11-28

**Authors:** Luca Brombal, Fulvia Arfelli, Pasquale Delogu, Sandro Donato, Giovanni Mettivier, Koen Michielsen, Piernicola Oliva, Angelo Taibi, Ioannis Sechopoulos, Renata Longo, Christian Fedon

**Affiliations:** 1 Department of Physics, University of Trieste, 34127 Trieste, Italy; 2 INFN Division of Trieste, 34127 Trieste, Italy; 3 Department of Physical Sciences, Earth and Environment, University of Siena, 53100 Siena, Italy; 4 INFN Division of Pisa, 56127 Pisa, Italy; 5 Department of Physics, University of Napoli Federico II, 80126 Fuorigrotta Napoli, Italy; 6 INFN Division of Napoli, 80126 Fuorigrotta Napoli, Italy; 7 Department of Radiology and Nuclear Medicine, Radboud University Medical Center, 6500 HB Nijmegen, The Netherlands; 8 Department of Chemistry and Pharmacy, University of Sassari, 07100 Sassari, Italy; 9 INFN Division of Cagliari, 09042 Monserrato Cagliari, Italy; 10 Department of Physics and Earth Science, University of Ferrara, 44122 Ferrara, Italy; 11 INFN Division of Ferrara, 44122 Ferrara, Italy; 12 Dutch Expert Center for Screening (LRCB), 6503 GJ Nijmegen, The Netherlands

**Keywords:** Biophysics, Biological physics

## Abstract

In this study we compared the image quality of a synchrotron radiation (SR) breast computed tomography (BCT) system with a clinical BCT in terms of contrast-to-noise ratio (CNR), signal-to-noise ratio (SNR), noise power spectrum (NPS), spatial resolution and detail visibility. A breast phantom consisting of several slabs of breast-adipose equivalent material with different embedded targets (i.e., masses, fibers and calcifications) was used. Phantom images were acquired using a dedicated BCT system installed at the Radboud University Medical Center (Nijmegen, The Netherlands) and the SR BCT system at the SYRMEP beamline of Elettra SR facility (Trieste, Italy) based on a photon-counting detector. Images with the SR setup were acquired mimicking the clinical BCT conditions (i.e., energy of 30 keV and radiation dose of 6.5 mGy). Images were reconstructed with an isotropic cubic voxel of 273 µm for the clinical BCT, while for the SR setup two phase-retrieval (PhR) kernels (referred to as “smooth” and “sharp”) were alternatively applied to each projection before tomographic reconstruction, with voxel size of 57 × 57 × 50 µm^3^. The CNR for the clinical BCT system can be up to 2-times higher than SR system, while the SNR can be 3-times lower than SR system, when the smooth PhR is used. The peak frequency of the NPS for the SR BCT is 2 to 4-times higher (0.9 mm^−1^ and 1.4 mm^−1^ with smooth and sharp PhR, respectively) than the clinical BCT (0.4 mm^−1^). The spatial resolution (MTF_10%_) was estimated to be 1.3 lp/mm for the clinical BCT, and 5.0 lp/mm and 6.7 lp/mm for the SR BCT with the smooth and sharp PhR, respectively. The smallest fiber visible in the SR BCT has a diameter of 0.15 mm, while for the clinical BCT is 0.41 mm. Calcification clusters with diameter of 0.13 mm are visible in the SR BCT, while the smallest diameter for the clinical BCT is 0.29 mm. As expected, the image quality of the SR BCT outperforms the clinical BCT system, providing images with higher spatial resolution and SNR, and with finer granularity. Nevertheless, this study assesses the image quality gap quantitatively, giving indications on the benefits associated with SR BCT and providing a benchmarking basis for its clinical implementation. In addition, SR-based studies can provide a gold-standard in terms of achievable image quality, constituting an upper-limit to the potential clinical development of a given technique.

## Introduction

Breast cancer is the leading cause of cancer death in women worldwide^1^. It is the most diagnosed cancer in women, accounting for one-third of all diagnosed cancers^2^.

The reference imaging technique for the early diagnosis of breast cancer is 2D digital mammography (DM). Recently, digital breast tomosynthesis (DBT), a pseudo 3D breast imaging modality^3^, has been developed to diminish the masking effect and the anatomical noise, showing potential applicability not only in the diagnostic domain but also in the screening setting^4–6^.

Another recent major technological advance is dedicated breast computed tomography (BCT), a fully 3D mammographic technique in which multiple low-dose projections are acquired and then reconstructed^7,8^. BCT is a relatively novel technique whose role is starting to be recognized in clinical practice^9,10^. BCT provides full 3D capabilities with adequate soft-tissue differentiation^11–15^. One of the big challenges for this technique is to combine the high resolution requirements and good lesion detection in a low-dose CT data acquisition^16,17^.

Presently, a number of dedicated BCT systems with different acquisition modes (e.g., cone-beam^11–15^, parallel-beam^18–20^, helical-CT^16,17,21^), imaging techniques (e.g., absorption^11–17^, phase-contrast^18–20,22^), and detector types (e.g., flat-panels^11–15,20^, photon-counting^16–19,21^), have been proposed. However, there is no study that directly compares the image quality metrics among different systems to date. Therefore, this study aims to provide the first quantitative comparison between a clinical BCT system^15^ and an under-development phase-contrast (PhC) synchrotron radiation (SR) BCT system^18,19^ based on a photon-counting detector, constituting a starting point for phantom-based image quality comparisons across different BCT systems.

Medical applications of SR have been successfully proven in both the diagnosis and therapy fields^23–26^. Of note, a PhC SR setup has already been applied in a mammographic clinical study^27^ demonstrating better image quality^28^ at lower radiation dose and with higher diagnostic power with respect to DM^29^; other clinically-oriented studies on breast-cancer diagnosis are presently underway at different synchrotron facilities^30,31^. Currently, the SYRMA-3D project^18,19^ at the SR facility Elettra (Trieste, Italy), is developing a PhC BCT system based on SR, combining high spatial resolution (by using a single-photon-counting detector^32^) and low delivered dose (by exploiting monochromatic highly-coherent SR). SR-based studies can provide a gold-standard in terms of achievable image quality (i.e., ideal imaging conditions) and they can constitute an upper-limit to the potential clinical development of a given technique. At the same time, assessing the difference with clinically available systems can provide a benchmark on the current level of behaviour of SR-based techniques, and therefore establish its potential for clinical implementation, as well as providing information relevant to the translational research aimed at developing compact high-coherence x-ray sources^33^. In this context, considering the lack of quantitative image-quality comparative studies, a comparison with the clinical domain is paramount.

Quantitative metrics such as contrast-to-noise ratio (CNR), signal-to-noise ratio (SNR), noise power spectrum (NPS), and spatial resolution are useful indicators of image quality, possibly related to diagnostic effectiveness^34^. Namely, CNR and SNR are related to low-contrast detail visibility (e.g., glandular tissue embedded in an adipose background), the shape of NPS reveals the image texture (i.e., low-frequency-peaked NPS is related to coarse image graininess; high-frequency-peaked NPS results in a finer grain noise), and spatial resolution determines the ability to detect small (high-contrast) details (e.g., microcalcifications).

The comparison between the two systems presented in this study is based on a breast-like phantom containing inserts mimicking relevant diagnostic features. The exposure parameters were automatically determined by the clinical BCT, while the SR irradiation parameters were tuned to replicate, as close as possible, the clinical conditions in terms of x-ray energy and delivered radiation dose.

## Materials and Methods

### Phantom

**Figure 1:**
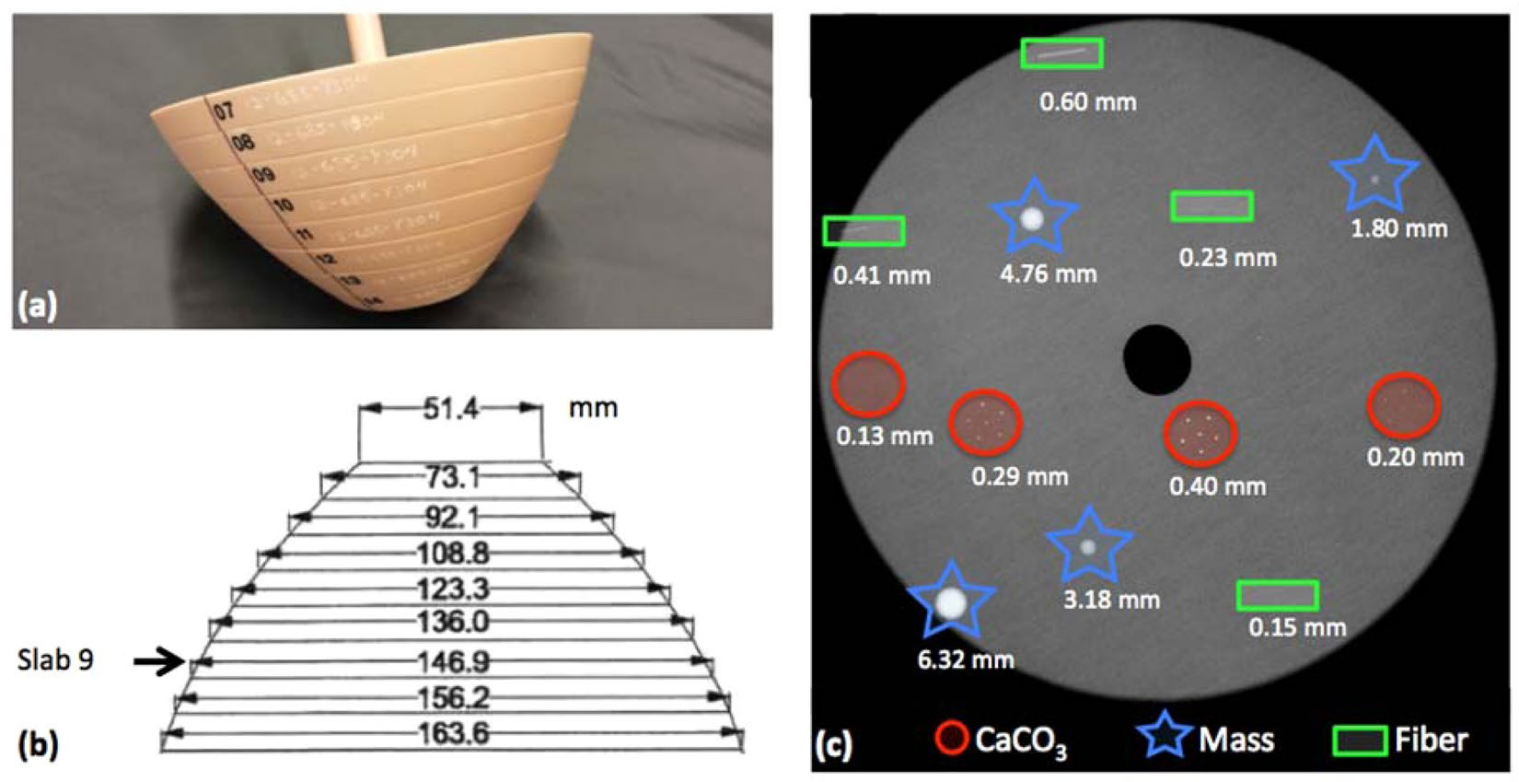
(**a**) Photograph of the phantom and (**b**) phantom dimensions [in mm]. (**c**) Details embedded in slab #9: calcifications (CaCO_3_) in red circles, masses in blue stars and fibers in green rectangles. The drawings are not to scale.

The custom-made breast phantom (design #12-685, CIRS, Norfolk, Virginia, USA) used to perform the imaging study is shown in Fig. 1.

The phantom has a semi-ellipsoidal truncated shape consisting of several slabs made of 100% breast-adipose equivalent material. A variety of targets are embedded into slab #9 as shown in Fig. 1c: spherical masses of different diameters (1.80 mm, 3.18 mm, 4.76 mm and 6.32 mm) made of epoxy resin with density equivalent to breast carcinoma; cylindrical fibers of different diameters (0.15 mm, 0.23 mm, 0.41 mm and 0.60 mm); and calcification clusters (CaCO_3_) of different grain sizes (0.13 mm, 0.20 mm, 0.29 mm, 0.40 mm). The lateral size of slab #9, ranging from 14 to 15 cm, corresponds to the typical mean breast diameter^35^. The phantom was positioned at the system isocenter both for the clinical BCT (see section 2.2) and for the SR BCT (see section 2.3).

### Clinical breast CT system

The dedicated breast CT clinical system^14,15,36–38^ (Koning Corp., West Henrietta, NY) evaluated in this work has a source-to-detector distance of 92.3 cm and a source-to-isocenter distance of 65.0 cm. The x-ray source (half-cone beam geometry) has a nominal focal spot size of 0.3 mm. The x-ray spectrum is tungsten target with aluminium filter set to a fixed voltage of 49 kV for all the acquisitions and a first half value layer of 1.39 mm Al (with a mean x-ray energy corresponding to 30.3 keV^15^, evaluated as the weighted-energy average of a photon spectrum model according to the work of Hernandez *et al*.^39^, based on air kerma measurements after attenuation by various thickness of Al). The x-ray tube operates in pulse mode, with a constant 8 ms pulse length. A complete BCT acquisition consists of 300 projections over a full 360° revolution of the x-ray tube and detector in 10 s. The appropriate tube current is selected by acquiring two low-dose projections (16 mA, 2 pulses of 8 ms each per projection) images at two orthogonal angles (i.e., 0 and 90 degrees).

The detector is a 39.7 cm × 29.8 cm flat-panel detector (4030CB, Varian Medical System, Palo Alto, California, USA) with a nominal pixel size of 194 µm, used in 2 × 2 binning mode. Tomographic reconstruction of the images is performed by using a Feldkamp-Davis-Kress (FDK)-based algorithm (with a modified Shepp-Logan reconstruction filter), with isotropic cubic voxels of 273 µm. The main system components and the phantom position can be found in the Supplementary Fig. S1. Measurements were performed positioning the phantom at the scanner isocenter.

The exposure parameter (i.e., the tube current) automatically selected by the clinical BCT for imaging the phantom determines the clinical settings and thus the mean glandular dose delivered, to which the SR photon’s fluence was tuned.

### Synchrotron radiation breast CT setup

The SR BCT system is under development at the SYRMEP (SYnchrotron Radiation for MEdical Physics) beamline at the SR facility Elettra (Trieste, Italy), within the framework of the SYRMA-3D project^18,19^.

The x-ray source of the SYRMEP beamline is produced by a bending magnet of the electron storage ring of Elettra. The source-to-detector distance is 31.6 m, while the source-to-isocenter distance is 30.0 m. Given the source spatial coherence and the 1.6 m isocenter-to-detector distance, this setup also allows for the exploitation of PhC effects, yielding, upon the propagation towards the detector, an increment in contrast across interfaces between different materials/tissue types^40^.

The SR beam is monochromatized by means of a Si (111) double-crystal monochromator providing monoenergetic x rays in the energy range of 9 keV to 40 keV, with an energy resolution ΔE/*E* = 2 × 10^−3^. A system of tungsten slits is used to define the beam shape, determining a rectangular cross section of 220.0 mm (horizontal)× 3.5 mm (vertical, Gaussian shape, FWHM) at the system’s isocenter (see Supplementary Materials Fig. S2). The small vertical dimension of the SR beam requires vertical translations of the patient support to perform fully three-dimensional tomographic acquisitions.

A system of calibrated ionization chambers^41^ provides the air kerma measurements necessary for the mean glandular dose evaluation^42^. The SR BCT system is equipped with a modular CdTe high-efficiency photon-counting x-ray imaging detector^43^ (PIXIRAD-8^32^) encompassing 8 modules covering a global active area of 246 × 25 mm^2^. The detector’s pixels are arranged on a honeycomb matrix with a pitch of 60 µm.

Acquired projection images undergo a detector-specific pre-processing procedure^44,45^. After data pre-processing, a phase-retrieval (PhR) algorithm^46^ is applied independently to each projection image. From a signal processing perspective, PhR is a specific low-pass filter that compensates for high-spatial frequency boost due to the free-space-propagation mechanism (i.e., edge enhancement), consequently reducing image noise^47^. As most low-pass filters, PhR can be tweaked to reach different trade-offs between image noise and spatial resolution^48^. As previously reported, phase-retrieval filters with two different parameter sets are applied to synchrotron-based images privileging either noise reduction or spatial resolution^48,49^. These two approaches are referred to smooth- and sharp-kernel PhR, corresponding to single (i.e., value of the parameter δ/β = 2267) and two materials (i.e., value of the parameter δ/β = 795), respectively, as specified in the work of Brombal *et al*.^48^.

Following the PhR procedure, projections are reconstructed via Filtered Back Projection (FBP) with Shepp-Logan filtering^50^ implemented via Graphics Processing Unit (GPU). Given the pixel’s honeycomb geometry and the slight magnification due to the beam divergence the reconstructed voxel size is 57 × 57 × 50 µm^3^.

Measurements were performed positioning the phantom at the system isocenter (see Supplementary Materials Fig. S2). The energy was set to 30 keV in order to match the average energy of the clinical BCT^14,15^, and the fluence was adjusted by means of aluminium filters to replicate the clinical dose level. Slab #9 was scanned in three vertical steps by acquiring 1200 projections over an angle of 180° per step, resulting in an overall scan time of approximately 100 s.

### Image quality analysis

The contrast-to-noise ratio (CNR) is defined by the ratio of the (average) pixel intensity difference between the  detail signal and the background ($\overline{{I}_{object}}$ *−* $\overline{{I}_{bkgd}}$), and the standard deviation of the background (*σ*_*bkgd*_) as follows^51^:

1 $$CNR=\frac{\overline{{I}_{object}}-\overline{{I}_{bkgd}}}{{\sigma }_{bkgd}}$$

Of note, the standard deviation of the background is assumed to represent the magnitude of the image noise (i.e., noise is assumed to be ergodic).

**Figure 2:**
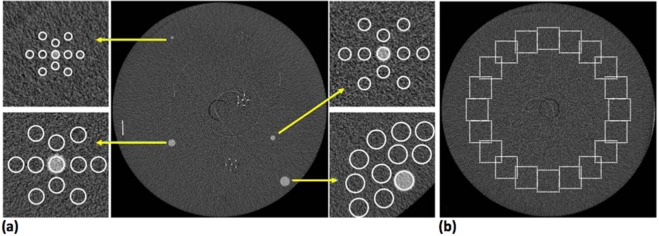
(**a**) Region of interest position for the contrast-to-noise ratio (CNR) and signal-to-noise ratio (SNR) evaluation. (**b**) Region of interest position for the noise power spectrum (NPS) evaluation in a homogeneous background.

With reference to the previous definition, CNR does not capture the dependence of detail visibility on the detail’s size (i.e., Rose criterion). Therefore, the signal-to-noise ratio (SNR_Rose_) metric can be introduced^52,53^:

2 $$SN{R}_{Rose}=CNR\times \sqrt{{N}_{pixel}}$$

where CNR is defined as in Eq. (1) and *N*_*pixel*_ is the number of pixels of the selected region of interest (ROI) within a given detail. For the sake of simplicity, hereafter the SNR_Rose_ is referred to as SNR. Both CNR and SNR were evaluated for all the spherical masses shown in Fig. 1c. For each mass a circular ROI with a diameter scaling with the mass dimension was selected within the detail, while, for the background estimation, 10 evenly spaced ROIs were selected in the neighbouring region (Fig. 2a), and the standard deviation was selected to be the average value of the background ROIs’ standard deviations. In the case of synchrotron-based datasets this analysis was repeated also by averaging 5 consecutive slices in order to match (as close as possible) the slice thickness of the clinical system, resulting in an effective voxel size of 57 × 57 × 250 μm^3^. With this choice a similar volume of a given detail is considered in each transverse slice for both systems.

While both CNR and SNR depend on the magnitude of the background noise, the image texture (or graininess) is characterized by the noise power spectrum (NPS), which is the noise spectral decomposition in the Fourier space. The in-slice NPS is a bi-dimensional map in Fourier space and it is measured from a homogeneous phantom CT image by selecting equally sized ROIs and using the following definition^54,55^:

3 $$NPS({f}_{x},{f}_{y})=\frac{{d}_{x}{d}_{y}}{{N}_{x}{N}_{y}}\frac{1}{{N}_{ROI}}\mathop{\sum }\limits_{i=1}^{{N}_{ROI}}{| {\mathcal F} [{I}_{i}(x,y)-{P}_{i}(x,y)]|}^{2}$$

where *f*_*x*_, *f*_*y*_ are the spatial frequencies, *d*_*x*_, *d*_*x*_ are the pixel sizes (mm) in *x* and *y* dimension, *N*_*x*_, *N*_*y*_ are the corresponding ROI dimensions measured in number of pixels, *N*_*ROI*_ is the number of selected ROIs, ${\mathcal F}$ denotes the bi-dimensional Fourier transform, *I*_*i*_ (*x*, *y*) is the pixel value at position (*x*, *y*) of the *i*-th ROI and *P*_*i*_ (*x*, *y*) is a 2^nd^ order polynomial fit of *I*_*i*_ (*x*, *y*). The subtraction with the polynomial term *P*_*i*_ (*x*, *y*) is a practical implementation of the de-trending procedure, aiming at removing any slowly-varying nonuniformities that may be caused from beam hardening effects, scattered radiation or nonuniform detector gain^55,56^.

As NPS is a spectral decomposition of image noise (σ), we have:

4 $${\sigma }^{2}={\iint }^{}NPS({f}_{x},{f}_{y})d{f}_{x}d{f}_{y}$$

Following the procedure described by Solomon *et al*.^55^, in order to compare only noise textures of images with different noise magnitudes, the normalized NPS (nNPS) is defined as:

5 $$nNPS({f}_{x},{f}_{y})=\frac{NPS({f}_{x},{f}_{y})}{{\sigma }^{2}}$$

In addition, since NPS maps of tomographic reconstructions usually show circular symmetry, it is common to show one-dimensional radially averaged NPS curves making use of the identity ${f}_{r}=\sqrt{{f}_{x}^{2}+{f}_{y}^{2}}$.

The nNPS distributions, both bi- and mono-dimensional, were evaluated for both systems by selecting 20 evenly spaced square ROIs at a constant distance from the phantom center as shown in Fig. 2b.

Given the difference in the reconstructed voxel size between the two systems, the used ROIs have a 64 × 64 pixels area for the clinical and system 256 × 256 pixels area for the synchrotron datasets, meaning that each ROI represents a similar physical area for both systems.

The uncertainty on radial nNPS curves was assessed by repeating the measure in 10 different homogeneous slices and associating, for each spatial frequency, the corresponding standard deviation^56^.

The spatial resolution of both systems was estimated starting from the reconstructed images by using a rather novel approach introduced by Mizutani *et al*.^57^, which is based on a logarithmic intensity plot in the Fourier domain, and it has shown consistent results for both planar and tomographic applications^58^. The main advantage of this technique is that it allows to estimate spatial resolution directly from general sample images, not requiring dedicated phantoms, under the hypothesis of a Gaussian system point spread function (PSF). Although modern digital detectors, especially direct conversion devices, in general do not feature Gaussian response functions, the whole imaging chain PSF contains also the contribution of each processing step leading to the final tomographic image. In particular, both the interpolation and the apodization filter inherent to tomographic reconstruction contribute to smoothen the system PSF^59^, usually described by a bell-shaped curve which, in the case of the presented technique, is approximated by a Gaussian function. Under this assumption, the full width at half maximum (FWHM) of the PSF can be determined from the following equation:

6 $$\mathrm{ln}\,{|{ {\mathcal F} }_{r}[I(x,y)]|}^{2}\cong -\frac{{\pi }^{2}}{2\,\mathrm{ln}(2)}FWH{M}^{2}{|{f}_{r}|}^{2}+constant$$

where ${ {\mathcal F} }_{r}[I(x,y)]$ is the radially averaged Fourier transform of the image* I(x,y)*. As input image* I(x,y)*, a wide square ROI enclosed within the uniform region of the phantom was selected, then $\mathrm{ln}\,{|{ {\mathcal F} }_{r}[I(x,y)]|}^{2}$ was plotted as function of ${|{f}_{r}|}^{2}$ and a low-frequency linear region was identified. According to Eq. (6) the slope of this region, extracted by a linear regression, is proportional to the square of the FWHM of the image PSF. Once the FWHM of the Gaussian PSF is known, the spatial resolution corresponding to the 10% of the modulation transfer function (MTF), measured in line-pairs per millimeter (lp/mm), can be easily estimated from^60^:

7 $$MT{F}_{10 \% }(lp/mm)=\frac{1}{1.24\times FWHM(mm)}$$

The presence of the factor 1.24 in the previous formula is justified in the Supplementary Materials (Eqs. (SE1) to (SE5)).

All the aforementioned quantitative analyses were performed developing a computer code in MATLAB (The MathWorks, Natick, MA, USA).

In addition, a qualitative analysis on the visibility of high-resolution details (i.e., calcification clusters and fibers) was performed by comparing the tomographic reconstructions of both setups.

## Results

The tube current automatically selected by the clinical BCT system for the breast phantom was 50 mA (for the 49 kV spectrum with a mean x-ray energy corresponding to 30.3 keV). This tube current value leads to an air kerma of 10.7 mGy and to a mean glandular dose^15^ of 6.5 mGy, for a breast diameter of 14 cm and a glandular fraction of 0%.

For the SR BCT, the air kerma was 14.2 mGy, corresponding to a radiation dose (i.e., MGD_v_ parameter evaluated according to Mettivier *et al*.^42^) of 6.7 mGy for a 14 cm breast diameter and a glandular fraction of 0%.

### Quantitative metrics (SNR, CNR, NPS and spatial resolution)

**Figure 3:**
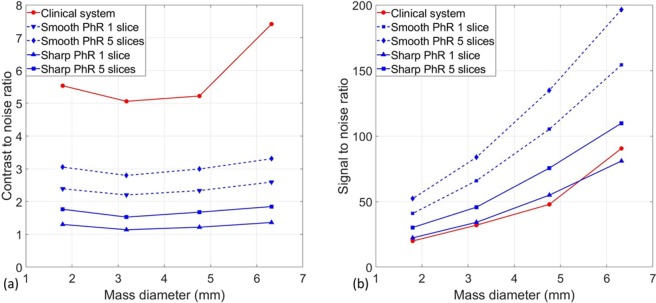
(**a**) Contrast-to-noise ratio and (**b**) signal-to-noise ratio as a function of mass diameter for the clinical breast CT (red solid line) and synchrotron breast CT with smooth (blue dashed lines) and sharp (blue solid lines) phase-retrieval kernels. Of note, in both plots, the point relative to the largest mass scanned with the clinical system produces a higher-than-expected CNR/SNR due to a reconstruction artifact (see Supplementary Materials Fig. S3).

Figure 3a shows the CNR values as a function of mass dimension for the two BCT systems (red colour for the clinical one and blue colour for the SR). In the case of SR images, the two PhR kernels and the two slice approaches (i.e., single slice and average over 5 consecutive slices to match the clinical slice thickness) are reported.

The CNR in the clinical BCT system is higher than in the SR case, regardless of the reconstruction and/or averaging methods: this is mainly due to the difference in the reconstructed voxel size. It has to be noted that an edge artifact in the phantom’s periphery involves the 6.32 mm mass leading to higher CNR, (see Fig. S3 in Supplementary Materials).

On the contrary, considering the detail visibility (Fig. 3b), which accounts for the number of pixels enclosed within the detail of interest, the synchrotron data show superior performance in all configurations, yielding, in case of the smooth PhR kernel and slice averaging, a 2.5 to 3 times higher SNR for all mass diameters.

**Figure 4:**
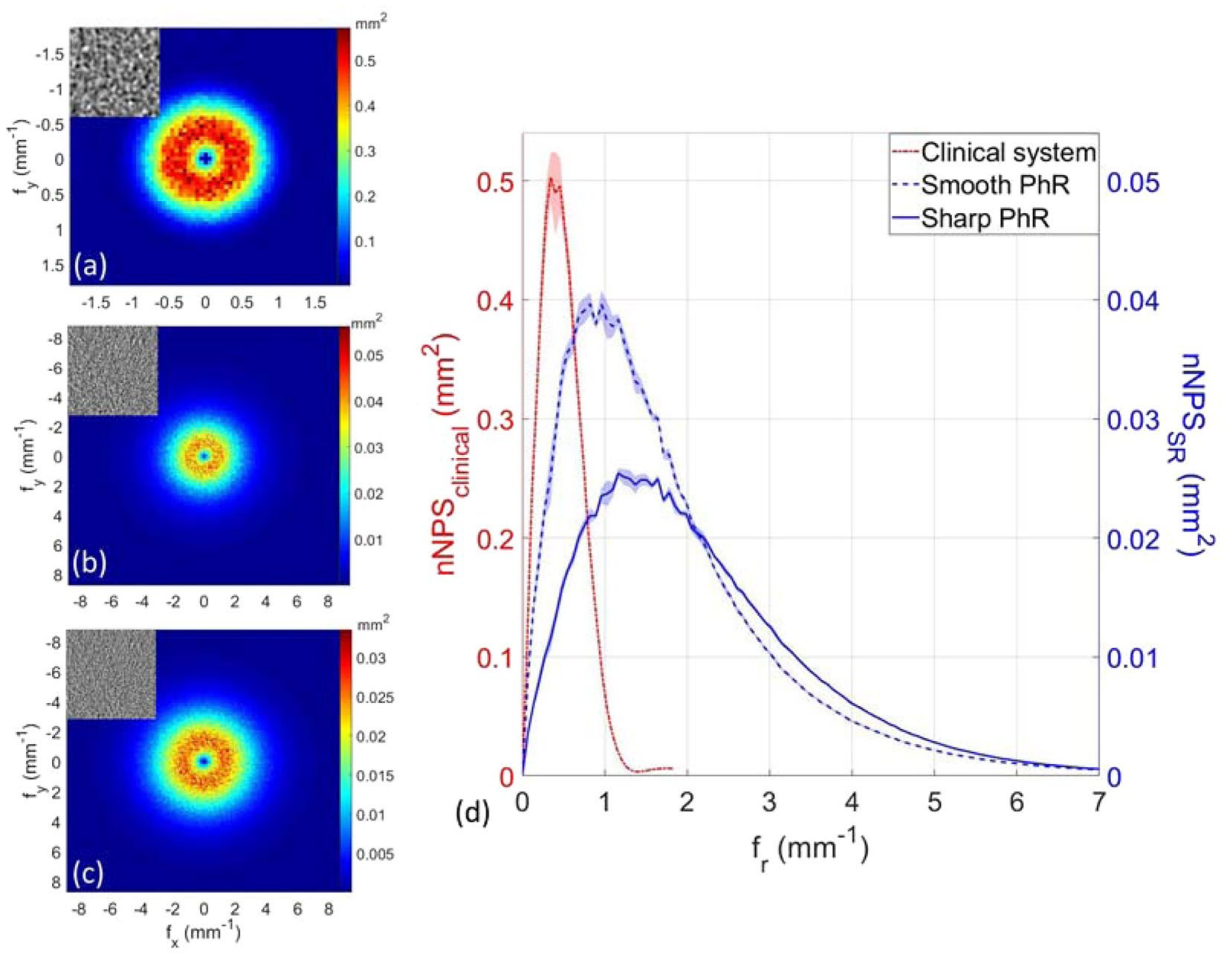
Bi-dimensional normalized noise power spectra (nNPS) for (**a**) the clinical BCT system, synchrotron BCT with (**b**) smooth and (**c**) sharp phase-retrieval algorithm. Of note, the range of frequency axes in (**a**) is different from (**b**,**c**). The inset on the top-left corner of each image represent the same homogeneous ROI with an area of 20×20 mm^2^. (**d**) Radial averaged nNPS for the clinical system (dashed red line), and SR breast CT with smooth (dashed blue line) and sharp (solid blue line) phase-retrieval algorithm. Of note, the left y-axis refers to the nNPS of the clinical system, while the right y-axis to the synchrotron breast CT. The shaded region around each line represents one standard deviation uncertainty.

Figure 4(a–c) show the bi-dimensional nNPS distributions for the clinical system and SR images with smooth and sharp PhR kernels. The noise in the clinical system is much coarser than in SR images, as visible in the upper insets of Fig. 4(a–c). Given the circular symmetry of bi-dimensional nNPS, their radial profiles were computed and plotted in Fig. 4d.

Peak frequencies largely differ when comparing the two systems, being 0.4 mm^−1^ for the clinical BCT, 0.9 mm^−1^ and 1.4 mm^−1^ for the synchrotron images reconstructed with smooth and sharp PhR, respectively. In addition, the nNPS drops to 5% of its maximum value at 1 mm^−1^ for clinical images, and at 5–6 mm^−1^ for SR datasets meaning that the roll-off slopes of nNPS curves are substantially different.

**Figure 5:**
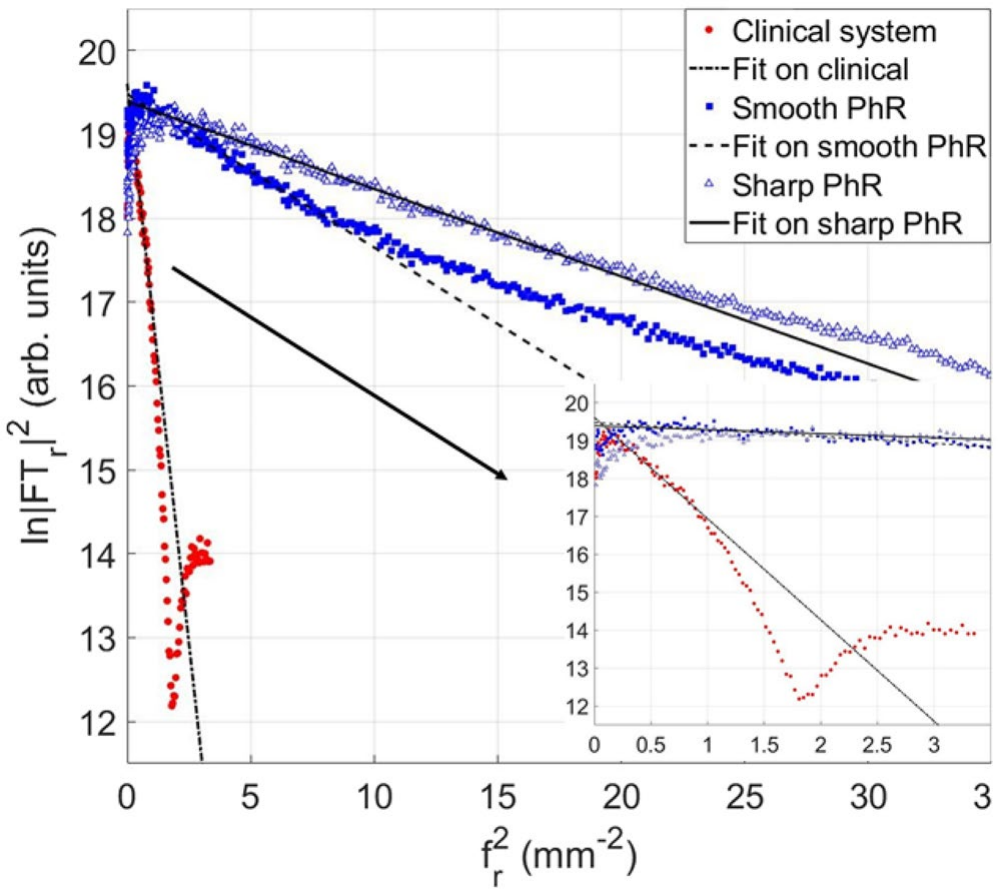
Evaluation of the spatial resolution for the clinical system (red circles), and synchrotron breast CT with smooth (blue squares) and sharp (blue-white triangles) phase-retrieval algorithm. The logarithm of the squared absolute value of the image Fourier transform (on y-axis) is plotted as function of the square of the spatial frequency (on x-axis). The linear fit for each dataset is shown with black lines. The inset displays a zoom at lower spatial frequencies.

The spatial resolution is estimated as shown in Fig. 5: a linear region at small spatial frequencies is identified for all the datasets, where steeper linear fits indicate worse spatial resolutions.

From the linear regressions (Eq. (6)), the spatial resolutions were estimated to be 0.61 mm (FWHM) or 1.3 lp/mm (MTF_10%_) for the clinical BCT, and 0.16 mm or 5.0 lp/mm (MTF_10%_) for the smooth PhR and 0.12 mm or 6.7 lp/mm (MTF_10%_) for the sharp PhR in SR images. The quantitative analysis results are summarized in Table. 1.

**Table 1:** Summary of the comparison analysis between the two systems: clinical BCT and synchrotron radiation (SR) datasets (with smooth and sharp phase-retrieval (PhR) kernel).

	CNR	SNR	nNPS peak frequency (mm^−1^)	FWHM (mm)	MTF_10%_ (lp/mm)
Clinical BCT	5.2	48	0.4	0.61	1.3
SR smooth PhR	2.3 (1 slice)	105	0.9	0.16	5.0
	3.0 (5 slices)	135			
SR sharp PhR	1.2 (1 slice)	55	1.4	0.12	6.7
	1.7 (5 slices)	76			

The table reports the values for contrast-to-noise ratio (CNR) and signal-to-noise ratio (SNR) for the 4.76 mm diameter mass, the peak frequency of the normalized noise power spectrum (nNPS), full width at half maximum (FWHM) of the estimated point spread function (PSF) and modulation transfer function at 10% (MTF_10%_)_._ CNR and SNR are measured by selecting 1 slice and the average of 5 slices, while the other values can be found in Supplementary Materials (Tab. S1).

### Qualitative analysis on lesion detectability

**Figure 6:**
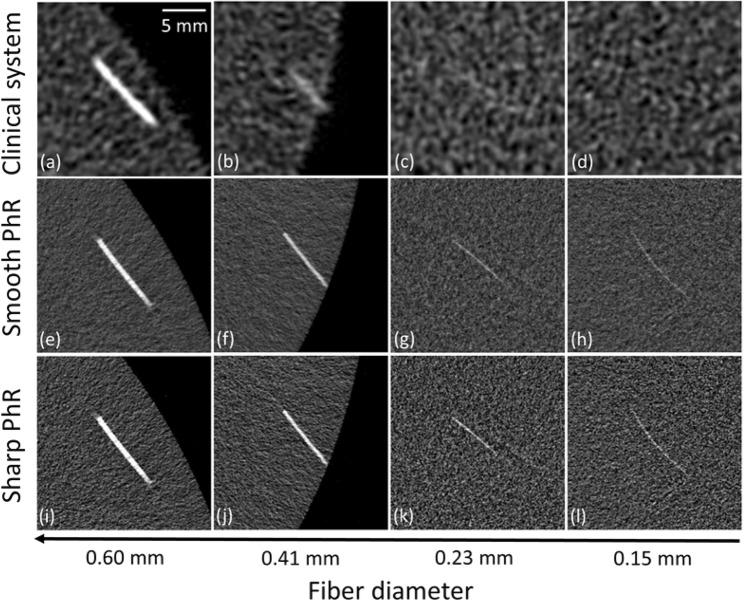
Details of the fibers reconstructed (**a**–**d**) with the clinical BCT system, (**e**–**h**) smooth and (**i**–**l**) sharp phase-retrieval (PhR) kernels for the synchrotron BCT. The scale reference is reported on the upper right corner of image (**a**).

Figure 6(a–l) display the epoxy fibers reconstructed from the clinical (a–d) and SR datasets with smooth (e–h) and sharp (i–l) PhR kernels. All the fibers are visible in the SR-based images regardless of the used PhR kernel, while the two smallest fibers (0.23 mm and 0.15 mm in diameter) are not distinguishable in clinical BCT images.

**Figure 7:**
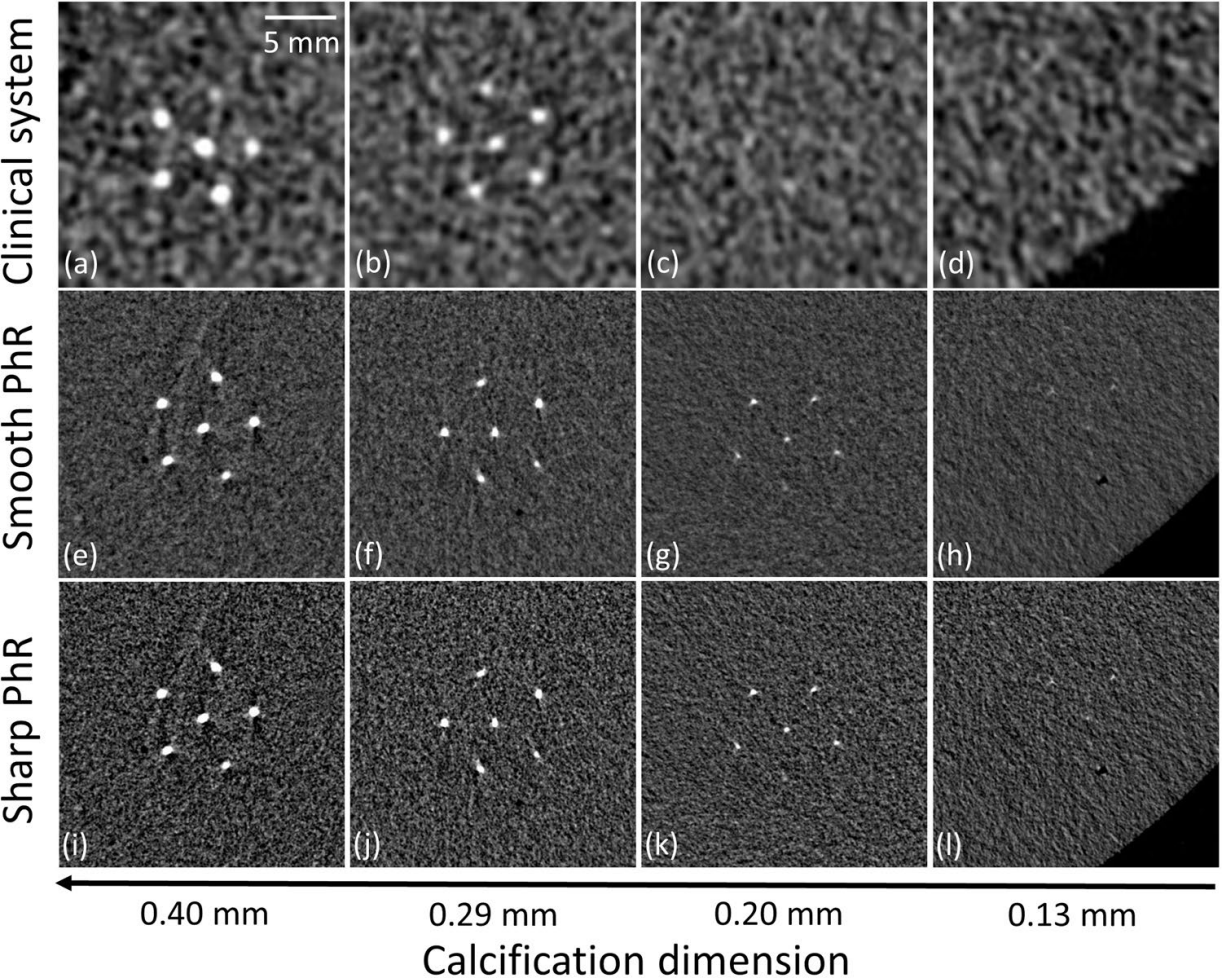
Details of the calcification clusters reconstructed (**a**–**d**) with the clinical BCT system, (**e**–**h**) smooth and (**i**–**l**) phase-retrieval (PhR) kernels for the synchrotron BCT.

Figure 7(a–l) show image details of the calcification clusters for the clinical (a–d) and SR datasets with smooth (e–h) and sharp (i–l) PhR kernels. For the clinical BCT system, no calcification cluster with diameter below 0.20 mm can be properly identified, while in the case of SR breast CT the smallest calcifications (0.13 mm in diameter) represent the visibility limit for both the smooth and sharp PhR kernels.

## Discussion

The proposed image quality analysis assessed the gap in image quality between the PhC SR BCT setup and the clinical BCT system.

In terms of detail visibility, the smallest fibres (i.e., diameter of 0.15 mm) and calcification clusters (i.e., diameter of 0.13 mm) are visible in the SR BCT, while details below 0.20 mm cannot be identified in the clinical BCT system (Figs. 6 and 7).

The CNR for the SR breast CT is found to be (as a first approximation) constant for different mass dimensions, with small fluctuations mainly due to different noise levels (Fig. 3a). In particular, the two masses (diameters of 3.18 mm and 4.76 mm) positioned closest to the center of the phantom show a slightly lower CNR with respect to the two located in the phantom’s periphery: this behaviour is compatible with the usual radial noise dependence observed in CT reconstructions (i.e., higher noise in the center, lower noise in the periphery). In agreement with results published in previous studies^48,49^, the smooth-kernel PhR yield a 2-fold higher CNR with respect to the sharp-kernel PhR. As mentioned, the higher CNR value observed for the mass of 6.32 mm in the clinical BCT images is caused by a reconstruction artifact close to the phantom’s edge where the mass is located (as reported in Supplementary Materials Fig. S3).

The SNR for the SR setup can be up to 3-times higher with respect to the clinical BCT if the smooth reconstruction kernel is used when the average of 5 slices is considered, or more than 2-times higher if no averaging is performed (Fig. 3b). This difference can be mainly attributed to the high-efficiency and low-noise of the photon-counting detector, to the presence of phase-contrast effects, which allows the phase-retrieval filter to be applied, and to the higher dose-efficiency of the synchrotron system due to the beam monochromaticity. In addition, thanks to the laminar shape of the beam and the large isocenter-to-detector distance, the SR setup allows to obtain inherently scatter-free images.

Considering SR-based data, it should be noted that, if the noise of each slice was uncorrelated, the expected SNR/CNR increase due to the averaging of 5 slices would be of a factor $\sqrt{5}$, whereas the observed factor is much smaller (between 1.3 and 1.4). This is mainly related to the application of the phase-retrieval that, being a 2D filter in the projections domain introduces a certain degree of correlation also between neighbouring pixels belonging to different rows of pixels, hence to different slices.

The nNPS evaluation revealed that the synchrotron images have a 2 to 4-times higher peak frequency (for the sharp phase-retrieval kernel, respectively) and a generally shallower roll-off slope, meaning that the contribution to the image noise is not negligible up to 6 mm^−1^, to be compared with 1 mm^−1^ of the clinical system’s case. It is worth noticing that the peak frequency for the clinical BCT (i.e., 0.4 mm^−1^) is consistent with previous findings of Betancourt-Benitez *et al*.^37^.

The observed differences in terms of nNPS between clinical and synchrotron data reveals that the SR setup imaging chain (i.e. detector, image processing and tomographic reconstruction) provides generally sharper or, equivalently, less correlated noise: this is ultimately related to the smaller detector pixel size and to the higher image-sharpness offered by direct-conversion photon-counting detectors.

Spatial resolution was estimated by using a rather novel technique that can be applied, in principle, to any tomographic image (under the approximation of a Gaussian-shaped system PSF), thus not needing a specifically designed phantom. Of note, since system PSFs are not in general described exactly by a Gaussian function, this method cannot fully replace the direct PSF and MTF measurements based on line-patterns or small high-absorbing details, but has to be regarded as a fast and easy way to provide a spatial resolution estimate or to be used for routine checks. The results obtained on the SR images, with both the smooth and sharp PhR kernels, are compatible with conventional spatial resolution estimates (based on the edge spread function technique) documented in previous studies^49,61^.

Remarkably, the spatial resolution estimated for clinical BCT through the Fourier space linear regression method (i.e., 1.3 lp/mm) is in reasonable agreement to the value found by Betancourt-Benitez *et al*.^38^ (i.e., ~1.1 lp/mm), by using the conventional tungsten-wire procedure.

Quantitatively, the spatial resolution of the SR system was found to be 4 to 5 times better than the clinical system (5 to 7 lp/mm for the synchrotron to be compared with 1.3 lp/mm for the clinical setup). Interestingly, synchrotron images outperform every clinical breast CT setup reported in literature so far^8^ in terms of spatial resolution, the maximum being 5 lp/mm for a photon-counting BCT system proposed by Kalender *et al*.^16,17,21^.

Nevertheless, it should be noted that the implementation of SR BCT to the clinical realm presents also some practical drawbacks. Besides the fact that SR facilities are limited in number, not allowing access to a wide population, SR BCT requires in general a longer scan time with respect to clinical systems due to the limited vertical dimension of the beam and to the need for patient rotation. This may lead to motion artifacts due to both voluntary and involuntary movements of the patient, possibly impairing image quality (mainly spatial resolution). This issue has been investigated also in a clinical context suggesting the use of a breast immobilizer^62^. In addition, the SYRMA-3D collaboration is devoting several efforts towards the reduction of the scan time, aiming to complete the examination in about 5 minutes^19^.

The metrics used in this work allow to show differences in terms of physical performances of the systems but not in their clinical impact (i.e., breast cancer detection and diagnosis), which should be evaluated through dedicated studies.

Of note, the selected SR beam energy replicates the average-output energy of the x-ray spectrum of the clinical BCT system without considering the effective energy shift due to beam hardening effect through the sample. However, recent results^63^ have shown that CNR, evaluated in glandular details embedded in a adipose background and fixed dose, has small variations (i.e., few percent) in the energy range between 30 and 37 keV, which includes the effective energy of the hardened spectrum for the clinical system.

Lastly, it should be stressed that, albeit SR BCT images rely not only on absorption contrast but also on phase effects, the application of the phase-retrieval algorithm produces images whose content describes the absorption properties of the sample^47,64^.

## Conclusions

This study compares the image quality difference between a BCT synchrotron radiation set-up using a photon-counting detector and a clinical BCT system. As expected, synchrotron-based images feature higher spatial resolution, SNR, and finer granularity; providing, for the first time, a quantitative assessment of this image quality gap.

It is clear that, despite offering remarkable performances, a widespread diffusion of SR BCT is not feasible in terms of costs and infrastructural requirements. In any case, SR-based studies can provide a gold-standard in terms of achievable image quality, constituting, in practice, an upper-limit to the potential clinical development of a given technique. Moreover, a direct assessment of the image quality improvements due to the use of synchrotron in comparison with commercially available setups is needed to substantiate the effort required to implement the BCT clinical program at Elettra synchrotron radiation facility. At the same time, the ongoing development of synchrotron-like compact x-ray sources may open up the possibility of exploiting the same techniques investigated in synchrotron facilities on a much wider domain.

## Supplementary information

Supplementary Materials

## Data Availability

The dataset analysed and discussed in the current study are available contacting the corresponding author on reasonable request.
