# Supplementary material for: Image quality comparison between a phase-contrast synchrotron radiation breast CT and a clinical breast CT: a phantom based study
Source: Sci Rep. 2019 Nov 28;9:17778. doi: 10.1038/s41598-019-54131-z (PMC6882794; doi:10.1038/s41598-019-54131-z)
Supplement: Supplementary file 1 — Supplementary Materials [file 41598_2019_54131_MOESM1_ESM.docx]

Image quality comparison between a phase-contrast synchrotron radiation breast CT and a clinical breast CT: a phantom based study

**Luca Brombal^1,2^, Fulvia Arfelli^1,2^, Pasquale Delogu^3,4^, Sandro Donato^1,2^, Giovanni Mettivier^5,6^, Koen Michielsen^7^, Piernicola Oliva^8,9^, Angelo Taibi^10,11^, Ioannis Sechopoulos^7,12^ , Renata Longo^1,2,*^, and Christian Fedon^7,2^**

^1^Department of Physics, University of Trieste, 34127 Trieste, Italy

^2^INFN Division of Trieste, 34127 Trieste, Italy

^3^Department of Physical sciences, Earth and Environment, University of Siena, 53100 Siena, Italy

^4^INFN Division of Pisa, 34127 Pisa, Italy

^5^Department of Physics, University of Napoli Federico II, 80126 Fuorigrotta, Napoli, Italy

^6^INFN Division of Napoli, 80126 Fuorigrotta, Napoli, Italy

^7^Department of Radiology and Nuclear Medicine, Radboud University Medical Center, 6500 HB Nijmegen, The Netherlands

^8^Department of Chemistry and Pharmacy, University of Sassari, 07100 Sassari, Italy

^9^INFN Division of Cagliari, 09042 Monserrato, Cagliari, Italy

^10^Department of Physics and Earth Science, University of Ferrara, 44122 Ferrara, Italy

^11^INFN Division of Ferrara, 44122 Ferrara, Italy

^12^Dutch Expert Center for Screening (LRCB), 6503 GJ Nijmegen, The Netherlands

*contact author: renata.longo@infn.ts.it

**
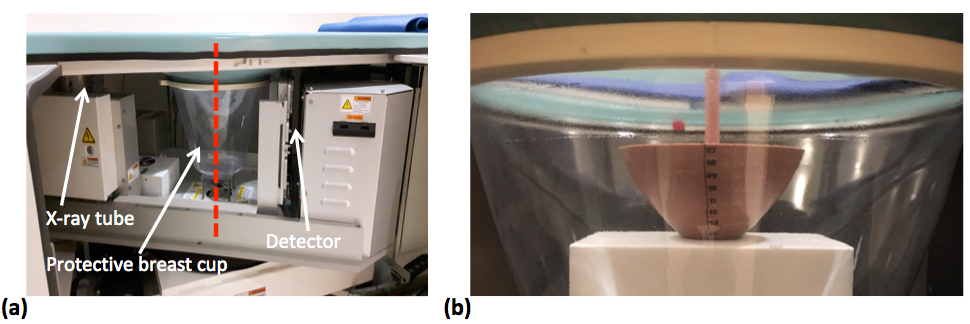
SUPPLEMENTARY MATERIALS**

**Figure S1.** (a) Photograph of the clinical breast computed tomography system components installed at the Radboud university medical center (Nijmegen, NL). The red dotted line represents the system isocenter. (b) Isocenter position of the phantom during the measurements.


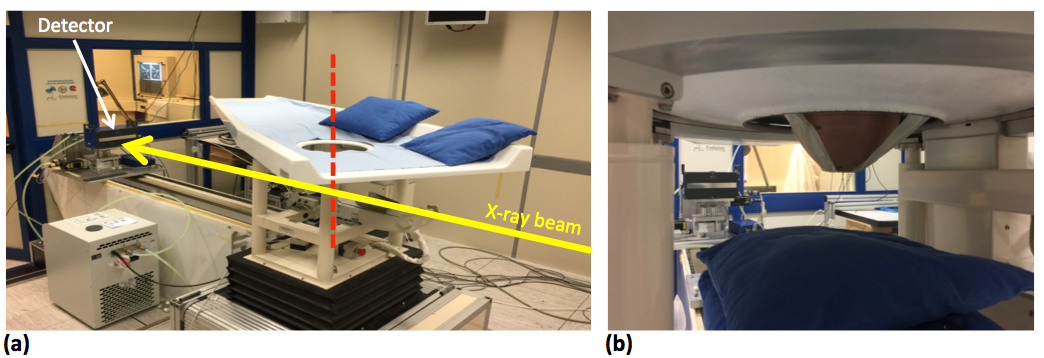
**Figure S2.** (a) Photograph of the synchrotron radiation breast computed tomography setup at the SYRMEP (SYnchrotron Radiation for MEdical Physics) beamline of the Elettra synchrotron facility (Trieste, Italy). The red dotted line represents the system isocenter. (b) Position of the phantom during the measurements.

**Equation SE1 – SE5.** Justification of the presence of the factor 1.24 in the formula (7)

Let’s consider a Gaussian PSF given by:

$PSF\left( x \right)=\frac{1}{\sigma\sqrt{2\pi}}\exp\left( -\frac{x^{2}}{2\sigma^{2}} \right)$ (SE 1)

and a FWHM given by:

$FWHM=2\sqrt{2ln(2)}\sigma$ (SE 2)

The corresponding MTF, function of the spatial frequency $f$, reads:

$MTF\left( f \right)=\left| \mathcal{F}\left[ PSF \right]\left( f \right) \right|=\exp\left( -\frac{\left( 2\pi f \right)^{2}\sigma^{2}}{2} \right)=exp \left( -\frac{\left( 2\pi f \right)^{2}FWHM^{2}}{2 \left( 2\sqrt{2\ln\left( 2 \right)} \right)^{2}} \right)$ (SE 3)

Where $\mathcal{F}$ denotes the Fourier transform. To find the spatial frequency corresponding to 10% of the MTF (i.e. $f_{10\%}$) means to invert the equation:

$10\%=\exp\left( -\frac{\left( 2\pi f_{10\%} \right)^{2}FWHM^{2}}{2 \left( 2\sqrt{2\ln\left( 2 \right)} \right)^{2}} \right)$ (SE 4)

Which results in:

$f_{10\%}=\frac{2}{\pi}\sqrt{\ln\left( 10 \right)ln(2)}\frac{1}{FWHM}\cong\frac{1}{1.24\times FWHM}$ (SE 5)

**Figure S3.** Close-to-edge artifact of the clinical BCT system reconstruction. As marked by yellow arrows in the right panels, the artifact has a radial symmetry and consists, when looking the phantom’s edge on the outward direction, of a thin hyper-intense region followed by a darker halo, potentially related to partial volume effects.

**
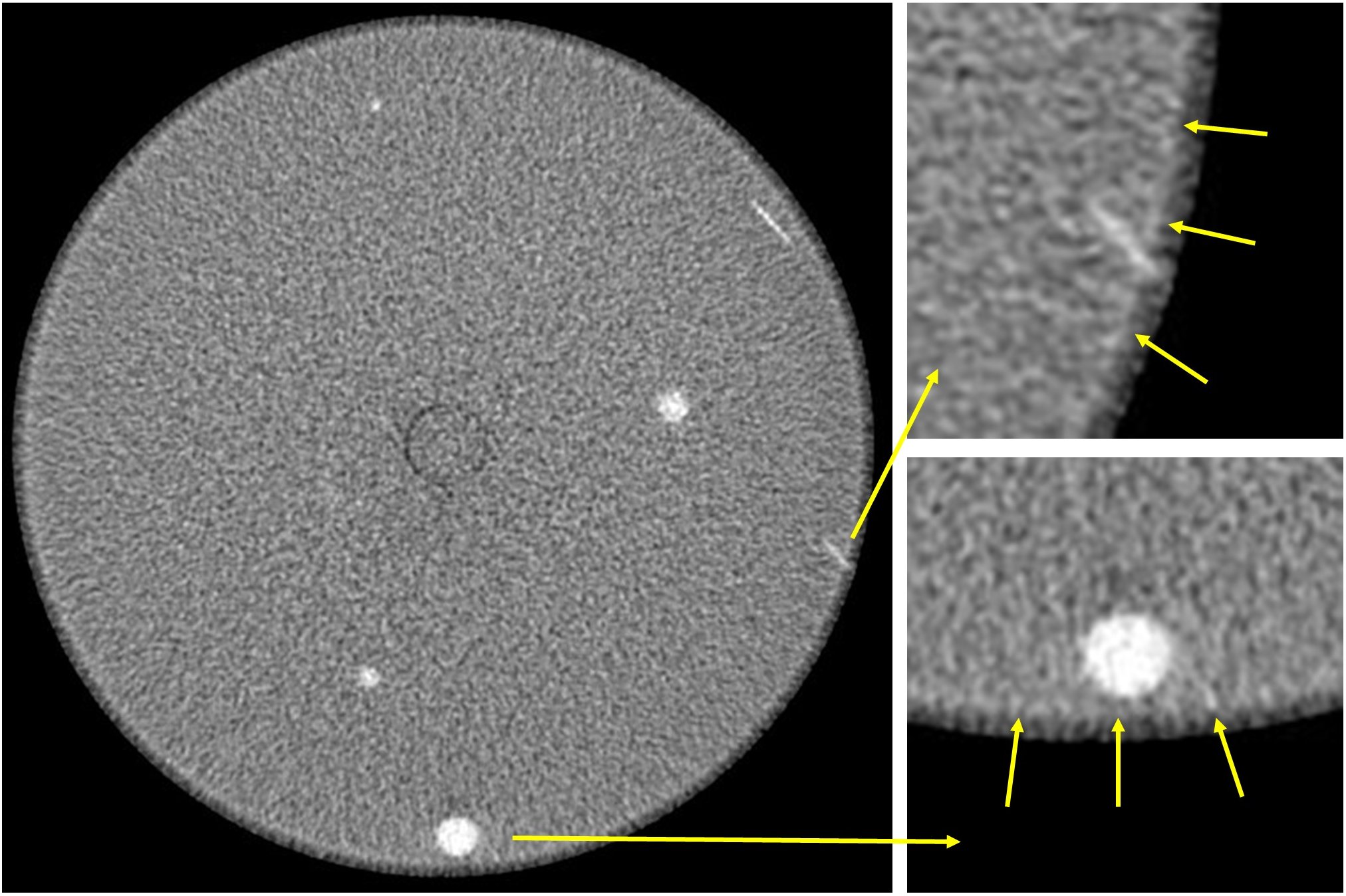
**

**Table S1.** Summary of the quantitative analysis of the phantom masses. For the synchrotron radiation (SR) datasets, both contrast-to-noise ratio (CNR) and signal-to-noise ratio (SNR) measured by selecting 1 slice and the average of 5 slices are reported.

|  | **CNR** | **SNR** |
| --- | --- | --- |
| 1.80 mm diameter mass | | |
| **Clinical BCT** | 5.5 | 20 |
| **SR smooth PhR** | 2.4 (1 slice) | 41 |
|  | 3.1 (5 slices) | 52 |
| **SR sharp PhR** | 1.3 (1 slice) | 22 |
|  | 1.8 (5 slices) | 30 |
| 3.18 mm diameter mass | | |
| **Clinical BCT** | 5.2 | 32 |
| **SR smooth PhR** | 2.2 (1 slice) | 66 |
|  | 2.8 (5 slices) | 84 |
| **SR sharp PhR** | 1.1 (1 slice) | 34 |
|  | 1.5 (5 slices) | 46 |
| 6.32 mm diameter mass | | |
| **Clinical BCT** | 7.4 | 91 |
| **SR smooth PhR** | 2.6 (1 slice) | 154 |
|  | 3.3 (5 slices) | 197 |
| **SR sharp PhR** | 1.4 (1 slice) | 81 |
|  | 1.8 (5 slices) | 110 |
